# Cell Culture Evaluation Hints Widely Available HIV Drugs Are Primed for Success if Repurposed for HTLV-1 Prevention

**DOI:** 10.3390/ph17060730

**Published:** 2024-06-05

**Authors:** Mphatso D. Kalemera, Allison K. Maher, Margarita Dominguez-Villar, Goedele N. Maertens

**Affiliations:** Department of Infectious Disease, Imperial College London, London W2 1PG, UK; m.kalemera@imperial.ac.uk (M.D.K.); a.maher19@imperial.ac.uk (A.K.M.); m.dominguez-villar@imperial.ac.uk (M.D.-V.)

**Keywords:** HTLV-1, integrase, INSTI, dolutegravir, reverse transcriptase, TAF, capsid, lenacapavir, vertical transmission, PrEP

## Abstract

With an estimated 10 million people infected, the deltaretrovirus human T-cell lymphotropic virus type 1 (HTLV-1) is the second most prevalent pathogenic retrovirus in humans after HIV-1. Like HIV-1, HTLV-1 overwhelmingly persists in a host via a reservoir of latently infected CD4^+^ T cells. Although most patients are asymptomatic, HTLV-1-associated pathologies are often debilitating and include adult T-cell leukaemia/lymphoma (ATLL), which presents in mature adulthood and is associated with poor prognosis with short overall survival despite treatment. Curiously, the strongest indicator for the development of ATLL is the acquisition of HTLV-1 through breastfeeding. There are no therapeutic or preventative regimens for HTLV-1. However, antiretrovirals (ARVs), which target the essential retrovirus enzymes, have been developed for and transformed HIV therapy. As the architectures of retroviral enzyme active sites are highly conserved, some HIV-specific compounds are active against HTLV-1. Here, we expand on our work, which showed that integrase strand transfer inhibitors (INSTIs) and some nucleoside reverse transcriptase inhibitors (NRTIs) block HTLV-1 transmission in cell culture. Specifically, we find that dolutegravir, the INSTI currently recommended as the basis of all new combination antiretroviral therapy prescriptions, and the latest prodrug formula of the NRTI tenofovir, tenofovir alafenamide, also potently inhibit HTLV-1 infection. Our results, if replicated in a clinical setting, could see transmission rates of HTLV-1 and future caseloads of HTLV-1-associated pathologies like ATLL dramatically cut via the simple repurposing of already widely available HIV pills in HTLV-1 endemic areas. Considering our findings with the old medical saying “it is better to prevent than cure”, we highly recommend the inclusion of INSTIs and tenofovir prodrugs in upcoming HTLV-1 clinical trials as potential prophylactics.

## 1. Introduction

There are few better indicators of the advances in medical care of the 20th century than our current abilities to treat and prevent infectious diseases [1]. Alongside improved sanitation and the advent of mass vaccination efforts, the availability of drugs to control infection has contributed enormously to improvements in humanity’s collective health. Nowadays, quick recovery with mild symptoms is the norm following diagnoses with some pathogens that would otherwise have meant certain death or physical disability less than a century ago. Human immunodeficiency virus (HIV) is one pathogen whose treatment has been radically transformed, exclusively by the advent of synthetic antiretroviral (ARV) drugs. Specifically, the single-pill regimen for combination antiretroviral therapy (cART) has provided a convenient option for patients, leading to improved adherence to therapy and, consequently, to sustained suppressed viraemia and a dramatic increase in life expectancy for those who promptly commenced treatment [2].

Perhaps owing to its endemic geographic distribution to non-western regions and long incubation period before the onset of fatality-associated disease, Human T-cell lymphotropic virus type 1 (HTLV-1), the first pathogenic human retrovirus to be identified, still has no approved therapeutic or preventative regimen [3]. However, the mortality and morbidity stemming from the HTLV-1-associated diseases acute T-cell leukaemia/lymphoma (ATLL) and HTLV-1-associated myelopathy/tropical spastic paraparesis (HAM/TSP) necessitate the development of effective clinical interventions. ATLL is an aggressive lymphoproliferative CD4^+^ malignancy seen in 5% of HTLV-1 carriers, for which there are limited treatment options, with median survival following diagnosis remaining stagnant at less than a year since the 1990s. Another 1–3% of HTLV-1^+^ individuals develop HAM/TSP, a blanket term for a group of neurodegenerative disorders that can leave patients wheelchair-dependent [4].

All retroviral genomes encode the enzymes reverse transcriptase (RT), integrase (IN) and protease (PR) [5]. RT converts single-stranded retroviral RNA genomes into double-stranded viral DNA (vDNA), which is then integrated into the host genome by IN to yield the provirus. Host-derived RNA polymerase and ribosomal machinery convert the provirus into viral polypeptides, which PR cleaves into mature, functional units. Of note, genetic and structural analyses indicate that the active site of each of these enzymes has a conserved architecture across all genera within the orthoretrovirinae subfamily [6,7,8]. Therefore, orthosteric synthetics developed for a specific virus within the subfamily, like HIV-1, are likely to be effective against orthoretroviruses from an evolutionary distant genus. To this end, there already exists a small body of literature demonstrating that all approved INSTIs tested against HTLV-1 so far, and the NRTI prodrug tenofovir disoproxil fumarate (TDF), potently prevent cellular transmission of the deltaretrovirus [9,10,11,12]. Indeed, the in vitro activity of tenofovir toward HTLV-1 was first described 20 years ago [13,14]. 

In the present work, we set out to assess the activity of recently approved drugs, or their components, against HTLV-1 infection in cell culture. A single combination ARV pill composed of the INSTI dolutegravir and the NRTIs emtricitabine and tenofovir alafenamide (DTG/FTC/TAF) developed by Viatris was approved by the US Food and Drug Administration as the first-line regimen for HIV-1 infection in developing countries in 2018 [15]. More recently, the European Medicines Agency (EMA) and the US Federal Drugs Administration (FDA) have approved lenacapavir (LEN), a capsid inhibitor, as a first-in-class, twice-yearly option for persons with multi-drug-resistant HIV-1 [16,17]. Here, we report that dolutegravir (DTG) and tenofovir alafenamide (TAF) both have potent activity against HTLV-1 in culture, with the former exhibiting an EC_50_ in the picomolar range, whereas LEN, expectedly, does not prevent HTLV-1 transmission. This work further underscores the clinical potential of INSTIs and tenofovir prodrugs in preventing HTLV-1 transmission.

## 2. Results

### 2.1. The NRTI Prodrug Tenofovir Alafenamide Inhibits HTLV-1 Transmission

Retroviral RT is a multifunctional enzyme. It harbors both DNA- and RNA-dependent DNA polymerase and RNase H activities, which enables the removal of genomic RNA from newly synthesized vDNA [18,19]. HIV RT is a heterodimer, with the active sites for both polymerase and RNase H located on the p66 subunit and the p51 subunit primarily serving a structural role. The NRTI tenofovir is an analogue of adenosine monophosphate. Mimicking nucleosides, NRTIs target the polymerase active site of RT, are incorporated into vDNA, and thus act as DNA chain terminators. 

**Figure 1:**
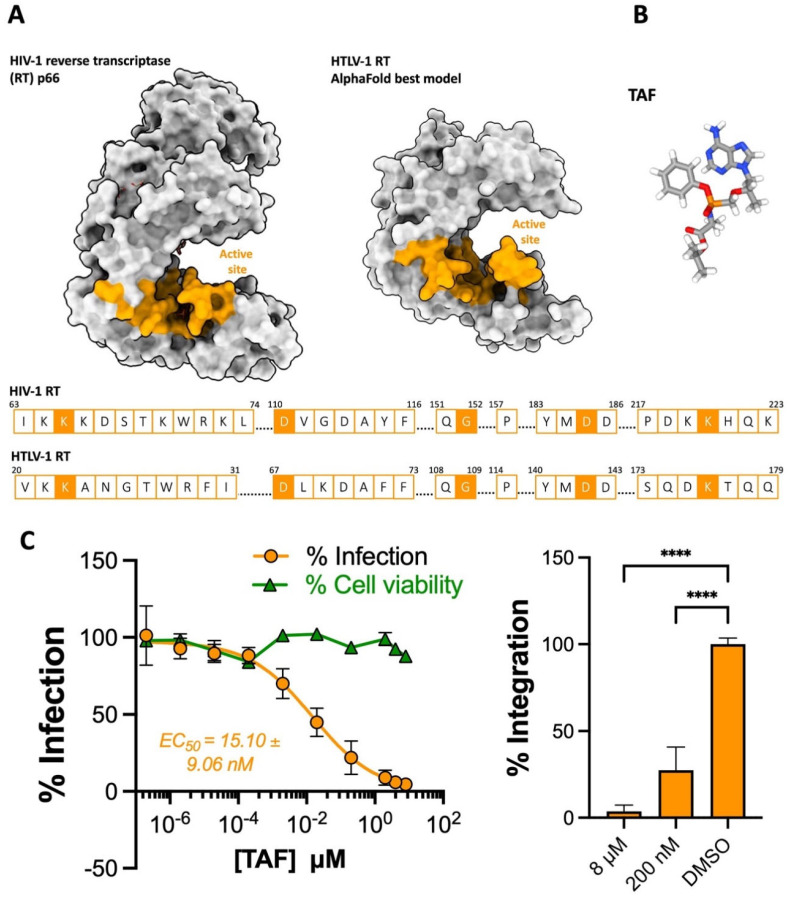
The activity of tenofovir alafenamide against HTLV-1 (**A**) Structures and active site sequence of HIV-1 and HTLV-1 reverse transcriptase (RT). Top panel: Surface structure of the HIV RT p66 monomer (PDB: 1T05) and the predicted structure of an HTLV-1 monomer generated using the AlphaFold protein prediction tool on ChimeraX. The active sites are colored in tangerine according to HIV-1 RT p66. Bottom panel: Alignment of residues in the active sites of HIV- and HTLV-1 RT, boxes filled in tangerine indicate residues identified to bind TAF in the 1T05 crystal structure. (**B**) Chemical structure of the prodrug TAF. (**C**) TAF blocks HTLV-1 transmission to Jurkat cells. Left panel—Jurkat cells were pre-treated with a dilution of TAF (10 concentrations starting from 8 μM two-fold down to 2 μM, then ten-fold down to 0.2 pM) and infected by coculture with MT-2 cells. Infection is represented as % of PVL relative to DMSO-treated cells, tangerine dots and curve. Cell viability is also expressed relative to DMSO-treated cells, green dots and line. Right panel—The percentage of integrated provirus of 8 µM, 200 nM and DMSO-treated Jurkat cells was determined relative to DMSO-treated samples. The data in C are from three independent experiments performed in triplicate. Error bars represent standard deviation. Asterisks denote statistical significance, following ordinary ANOVA using Dunnett’s multiple comparison test with the DMSO-treated samples as the control (**** *p* < 0.0001).

It is worth noting that although we lack a crystal structure of an active unit of HTLV-1 RT, both the alignment of HIV-1 and HTLV-1 RT amino acid sequences and the predicted model of a complete HTLV-1 RT monomer suggest that the active sites of the two enzymes are architecturally very similar (Figure 1A) [18]. Our past observation that the first prodrug formulation of tenofovir, TDF, effectively inhibits HTLV-1 culture transmission (EC_50_ = 17.78 ± 7.16 nM) supports this notion [9]. TDF is hydrolyzed by gut and plasma esterases into active tenofovir, meaning the active drug is present in plasma for protracted periods post-administration, increasing the chances of off-target exposure [20]. Conversely, the updated prodrug formula of tenofovir, TAF, is metabolized into active tenofovir intracellularly by cathepsin A, thus limiting its presence in plasma. This contributes to its improved safety profile and extended bioavailability within cells compared to the former [20]. Here, we assessed the anti-HTLV-1 activity of TAF (Figure 1B) by coculturing irradiated persistently infected MT-2 cells with target Jurkat cells in a range of concentrations of the prodrug. Following the depletion of MT-2 cells, de novo infection in Jurkat cells was quantified by determining the PVL following their expansion. TAF was observed to efficiently block HTLV-1 transmission without affecting cell viability in the range of concentration tested, achieving an EC_50_ of 15.10 ± 9.06 nM (Figure 1C, left panel). Although this figure is five times the average observed for a panel of 29 HIV-1 isolates [21], it nonetheless indicates that TAF possesses quite potent anti-HTLV-1 activity. Additionally, the highest concentration of TAF almost completely blocked HTLV-1 integration when quantifying HTLV-1 integration products following Alu-PCR amplification (Figure 1C, right panel). 

### 2.2. The 2nd Generation INSTI Dolutegravir Potently Inhibits HTLV-1 Transmission

Retroviral IN catalyzes the requisite insertion of vDNA into host chromosomal DNA, a defining step that sets retroviruses apart from other viral families [6]. IN engages the long terminal repeats (LTRs) at both termini of newly synthesized vDNA, forming a complex termed the intasome, and performs two enzymatic reactions. First, IN hydrolyses vDNA to remove two or three nucleotides following the invariant CA-dinucleotide, generating nucleophilic 3′-hydroxyl groups. Secondly, IN uses the reactive 3′-hydroxyl groups to cut both strands of chromosomal DNA and insert both 3′ ends of vDNA into the host genome [22]. The active site for both reactions is contained exclusively within the catalytic core domain of the enzyme. This domain harbors a DDE catalytic triad, and these residues coordinate a pair of functionally critical Mg^2+^ cations. By binding this divalent cation, INSTIs displace the vDNA end from the integrase active site and, in doing so, deactivate the intasome [23,24].

**Figure 2:**
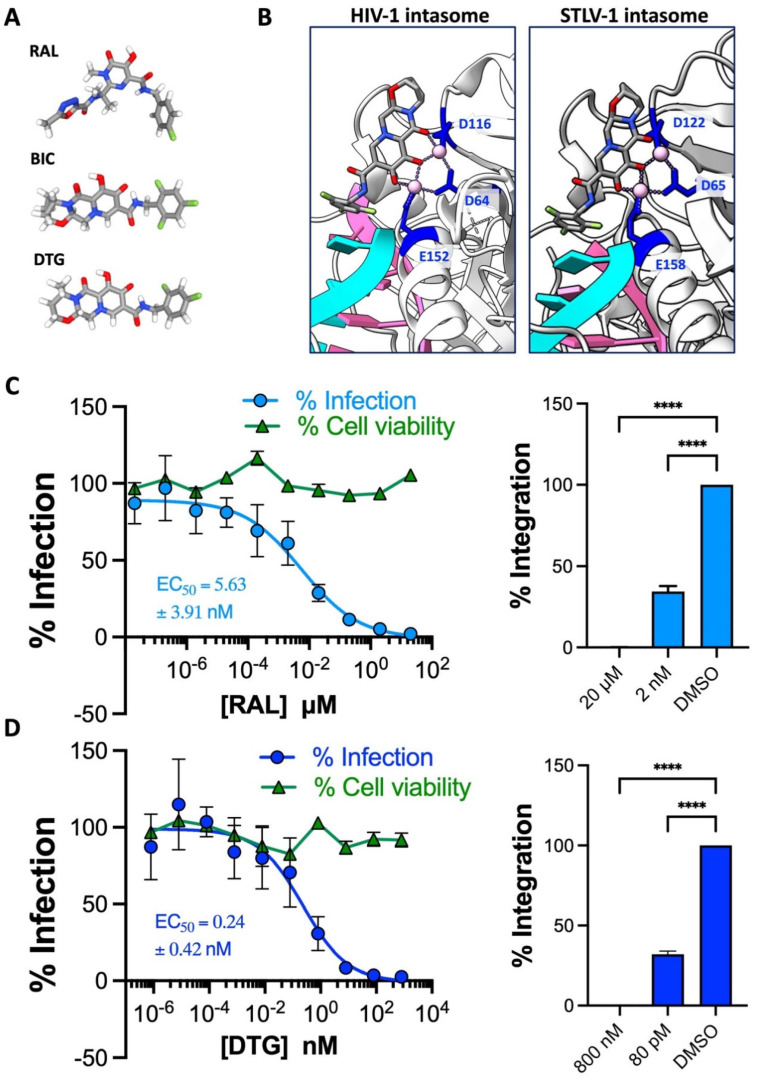
The activity of dolutegravir against HTLV-1. (**A**) Chemical structures of the 1st-generation INSTI raltegravir (RAL) and the 2nd-generation INSTIs bictegravir and dolutegravir (DTG). (**B**) View of the HIV-1 (PDB: 6PUW) and STLV-1 (7OUF) intasome active sites bound to bictegravir, vDNA chains are colored in cyan and pink. Residues comprising the DDE catalytic triad are highlighted in blue, with their sidechains displayed as sticks. Pink dots represent the critical Mg^2+^ cation pair. (**C**) RAL blocks HTLV-1 transmission to Jurkat cells. Left panel Jurkat cells were pre-treated with a serial dilution of RAL (10 concentrations starting from 20 μM log-diluted down to 20 fM) and infected by coculture with MT-2 cells. Infection is represented as % of PVL relative to DMSO-treated cells, sky blue dots and curve. Cell viability is also expressed relative to DMSO-treated cells, green dots and line. Right panel—The percentage of integrated provirus of 20 µM, 2 nM and DMSO-treated Jurkat cells was calculated relative to DMSO-treated samples. (**D**) DTG potently blocks HTLV-1 transmission to Jurkat cells. Left panel—Jurkat cells were pre-treated with a serial dilution of DTG (10 concentrations starting from 800 nM log-diluted down to 0.8 fM) and infected by coculture with MT-2 cells. Infection is represented as % of PVL relative to DMSO-treated cells, blue dots and curve. Cell viability is also expressed relative to DMSO-treated cells, green dots and line. Right panel—The percentage of integrated provirus of 800 nM, 80 pM and DMSO-treated Jurkat cells was calculated relative to DMSO-treated samples. Data in (**C**,**D**) are from three independent experiments performed in triplicate. Error bars represent standard deviation. Asterisks denote statistical significance, following ordinary ANOVA using Dunnett’s multiple comparison test with the DMSO-treated samples as the control (**** *p* < 0.0001).

Our previous work revealed approved INSTIs to be similarly potent in blocking HIV-1 and HTLV-1 cell culture transmission [9]. In later structural work [10,25], we observed that INSTIs occupy the active sites of HIV-1 and STLV-1 intasomes in virtually identical conformations (Figure 2B), providing mechanistic insights into the high sensitivity of HTLV-1 to INSTIs, which are derivatives of metabolites isolated for their anti-HIV-1 IN activity [26,27,28]. 

A concerning link between the second-generation INSTI DTG and neural-tube defects in Botswanan newborns was reported in 2018; consequently, we excluded this drug from bygone cell culture evaluation [29]. However, recent pharmacovigilance analyses did not uphold the link, dispelling safety concerns [30]. So, we set out to determine whether DTG is as potent as other INSTIs against HTLV-1 transmission in the present work. We first reconfirmed the sensitivity of HTLV-1 to the first-in-class INSTI raltegravir (RAL) in cell culture to ensure that comparisons between the new and past data sets are appropriate. As before, RAL strongly inhibited HTLV-1 transmission and showed no signs of cytotoxicity at the range of concentration tested (Figure 2C, left panel). An EC_50_ of 5.63 ± 3.91 nM was calculated from the dose–response curve and is consistent with the previously observed value of 6.42 ± 4.24 nM [9]. As observed for the other second-gen INSTIs bictegravir (BIC) and cabotegravir (CAB), DTG more potently prevented HTLV-1 transmission than RAL (Figure 2D, left panel). Fittingly, the EC_50_ of 0.24 ± 0.42 nM is in the same ballpark as those calculated for bictegravir and CAB [9,10,11]. Furthermore, we were unable to amplify any HTLV-1 integration products from Jurkat cells challenged with the highest concentration of RAL or DTG during coculturing with MT-2 cells, indicating the complete blockade of integration and, by extension, infection (Figure 2C,D, right panels). Notably, the EC_50_ values we report here are similar to those typically observed for both lab-adapted and clinical HIV-1 and HIV-2 isolates, suggesting INSTIs are equally potent toward HTLV-1 and HIV [31].

### 2.3. The HIV-1 Capsid Inhibitor Lenacapavir Is Inactive against HTLV-1

**Figure 3:**
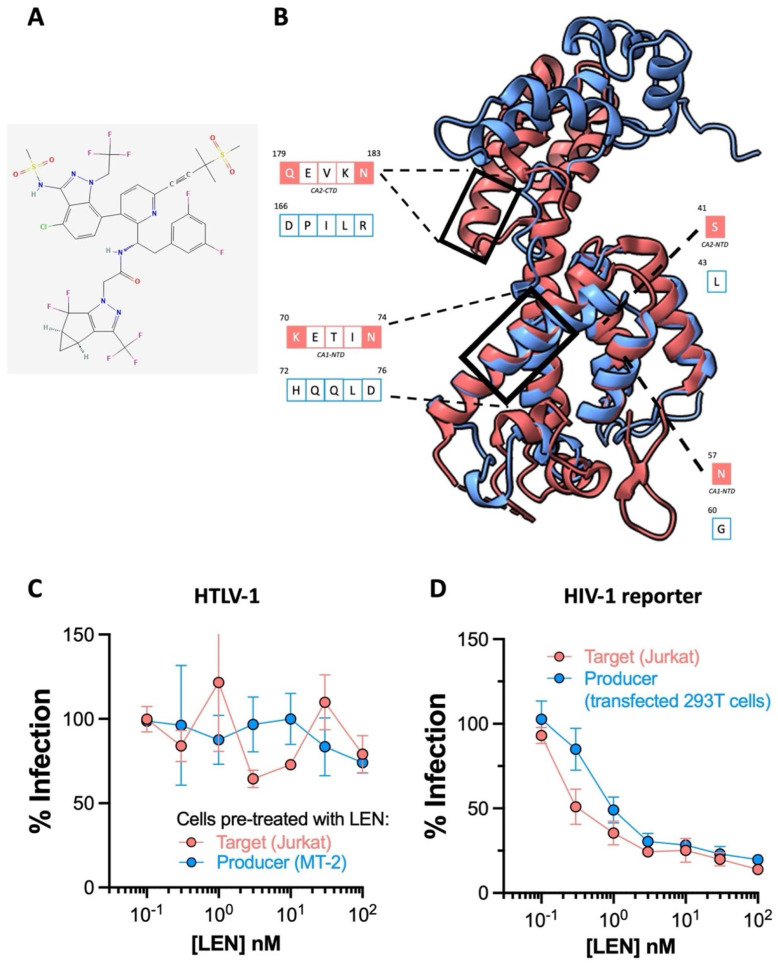
The activity of lenacapavir against HTLV-1. (**A**) Chemical structure of the HIV-1 capsid targeting lenacapavir (LEN). (**B**) The predicted structure of an HTLV-1 p24 colored in blue generated on AlphaFold superimposed onto HIV-1 p24 colored in salmon (PDB: 1E6J) using ChimeraX visualization software. The residues involved in HIV-1 p24-LEN binding are displayed in the boxes with salmon outlines. Filled boxes in salmon indicate residues that bind LEN. Corresponding residues in HTLV-1 p24 following superimposition or alignment are displayed in the boxes with blue outlines. (**C**) LEN is ineffective at blocking HTLV-1 transmission to Jurkat cells. Salmon points: Jurkat cells were pre-treated with a serial dilution of LEN (7 concentrations from 100 nM diluted 3-fold down to 140 pM) and infected by coculture with MT-2 cells to measure anti-HTLV-1 activity in the early stages of viral replication. Blue points: MT-2 cells were pre-treated with a serial dilution of LEN before coculture with Jurkat cells to measure anti-HTLV-1 activity in the late stages of viral replication. Infection is represented as % of PVL relative to DMSO-treated cells. (**D**) LEN potently blocks HIV-1 cell-free (VSV-G) pseudotyped infection of Jurkat cells. LEN is active against HIV-1 when pre-treated with both producer cells and target cells, as previously demonstrated. Data in (**C**,**D**) are from two independent experiments performed in triplicate. Error bars represent standard deviation.

Targeting the trifecta of retroviral enzymes has transformed the lives of HIV-1/2^+^ patients the world over. However, the emergence of multidrug-resistant strains as contributors to cART failure and the necessity of daily administration for current therapy impelled pharmaceutical companies to search for new amenable targets on the HIV polypeptide [32]. For Gilead Sciences, this quest culminated in developing lenacapavir (LEN) (Figure 3A) [33]. This drug functions by tightly stabilizing adjoining capsid/p24 subunits, thereby preventing the disassembly of the curve capsid lattice, and interfering with capsid association to essential HIV-1 cellular cofactors [34]. Of note, LEN is active at both the early and late steps of the HIV-1 life cycle, antagonizing HIV-1 replication at the pre-integration and pre-egress stages, respectively.

Striking findings from phase I clinical trials (NCT037339866C) suggest that six-month dosing intervals with LEN could effectively suppress HIV-1. Preexposure prophylaxis (PrEP) that can be administered twice yearly is poised to be clinically more attractive than that requiring daily administration. To evaluate whether LEN can prevent HTLV-1 transmission, we first generated a model of HTLV-1 p24 using the AlphaFold protein prediction tool on ChimeraX, since its crystal structure is yet to be solved. While superimposition implied the four alpha helices composing the N terminal domain of the best model of HTLV-1 p24 overlapped significantly with HIV-1 p24 (1E6J), structure-guided alignment revealed poor residue conservation in the helices binding LEN (Figure 3B). Consistent with this, the results from our cell culture experiments dismiss all prospects for adopting LEN as an HTLV-1-preventative intervention. We preincubated either the HTLV-1 producer MT-2 cells or target Jurkat cells with LEN to determine if the drug could block HTLV-1 at the pre-egress and pre-integration stages, respectively, and witnessed no discernible antiviral activity in either scenario (Figure 3C). Equivalent experiments with GFP reporter HIV-1 pseudoparticles confirmed this batch of LEN was active (Figure 3D). Here, we preincubated target Jurkat cells or 293T cells transfected to produce HIV-1 reporter virus with LEN, and expectedly observed the drug is potently active against HIV-1 at both the early and late stages, as previously reported [17].

### 2.4. TAF-, RAL- and DTG-Treated Primary CD4^+^ T Cells Resist HTLV-1 Infection

There is some heterogeneity in how susceptible individuals are to viral infections, a phenomenon that cannot be replicated in cell lines [35]. This variation is influenced by several factors, including polymorphisms in MHC alleles, mutations in genes that control innate receptors and cell-intrinsic restriction factors, and differences in cytokines, chemokines, and their receptors [35,36]. This heterogeneity may influence the efficacy of antiviral therapeutics and prophylactics in the population. To address this, we made use of the fact that CD4^+^ lymphocytes, the primary cellular targets of HTLV-1, are easily isolated from peripheral blood mononuclear cells and display extensive individuality and variation in gene expression [37]. This makes them suitable for testing whether heterogeneity may affect the efficacy of HIV drugs when repurposed for HTLV-1 prevention. Therefore, we conducted a study to test whether the compounds showing anti-HTLV-1 activity in the Jurkat cells could similarly prevent HTLV-1 transmission in primary CD4^+^ T cells isolated from the peripheral blood mononuclear cells of six healthy donors. Untouched CD4^+^ T cells were enriched using a commercially available kit and then treated with TAF, RAL, and DTG at two concentrations for three hours before coculture with irradiated MT-2 cells.

**Figure 4:**
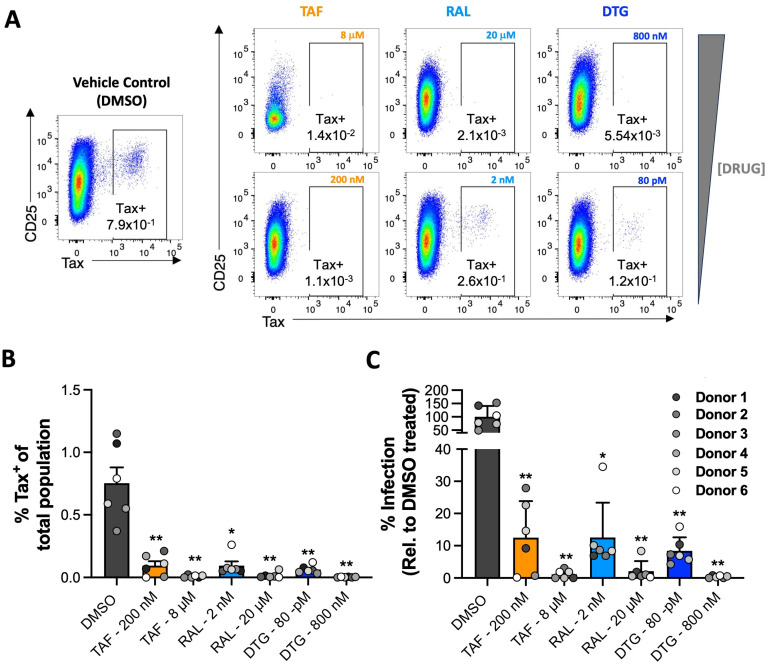
INSTIs and tenofovir prevent HTLV-1 transmission to primary CD4^+^ T cells in cell culture. Freshly isolated primary CD4^+^ T cells from 6 healthy donors were treated with two concentrations of TAF (tangerine), RAL (sky blue), and DTG (blue) for three hours before infection by coculture with irradiated MT-2 cells. Infection was quantified 12 days later via flow cytometry by staining for the HTLV-1 protein Tax. (**A**) Representative flow dot plots for the expression of CD25 and HTLV-1 Tax in samples from donor 6. (**B**) Bar graph collating data from all six donors. Data are expressed as % Tax^+^ cells of the total population acquired on the flow instrument. (**C**) Bar graph representing % infection relative to vehicle (DMSO)-treated samples. Each symbol in (**B**,**C**) represents one donor. Error bars represent standard deviation. Asterisks denote statistical significance, following ordinary ANOVA using Dunnett’s multiple comparison test with the DMSO-treated samples as the control (* *p* < 0.05, ** *p* < 0.01). Data represent a pool of two independent experiments with three donors each.

We quantified infection 12 days later via flow cytometry by staining for the HTLV-1 protein Tax, whose expression, as we recently reported, correlates strongly with the qPCR-determined proviral loads of CD4^+^ T cells from HTLV-1^+^ patients and, as such, represents a reliable non-qPCR-based readout for infection [38]. Gating for Tax^+^ cells indicated that we infected 0.5–1% of CD4^+^ T cells in DMSO (vehicle)-treated samples from all six donors. The complete or near-complete blockade of HTLV-1 transmission to primary T cells from all six donors was observed at the highest concentration of each drug tested, as indicated by the absence of Tax^+^ cells in these samples (Figure 4A—top row and Figure 4B). Furthermore, we observed minimal HTLV-1 transmission in samples treated with drug concentrations near the inflexion points of the dose–response curves generated from Jurkat cell experimentation (Figure 4A—bottom row). These findings support our results for Jurkat cells and illuminate the high efficacy with which these drugs prevent the HTLV-1 infection of ex vivo CD4^+^ T cells. Importantly, these results suggest that the in vivo anti-HTLV-1 activities of all three compounds are unlikely to be affected by host variability since we saw no differences in donor susceptibility to HTLV-1 infection at the high drug doses. Moreover, the high concentrations we use are only small fractions of the protein-adjusted concentrations observed in the blood of healthy persons taking PrEP (TAF) or cART (RAL and DTG) daily [39,40]. 

## 3. Discussion

A recent meta-analysis of epidemiological studies has suggested that HTLV-1^+^ status may be linked to at least a dozen diseases, including seborrheic dermatitis and Sjogren’s syndrome, in addition to the previously identified fatality- and myelopathy-associated pathologies [41]. Even asymptomatic patients have reported higher incidences of malaise, discomfort, and depression compared to the general population [42]. The reduced quality of life, the risk of developing currently untreatable ATLL and HAM/TSP, and the likely underestimation of the population living with HTLV-1 all highlight the urgent need for prophylactics and therapeutics in the clinic. Most prophylactic research efforts have understandably been directed towards developing vaccine candidates for HTLV-1, but none have advanced beyond small animal trials. Likewise, curative or genuinely transformative therapeutics have yet to be discovered for either of the main HTLV-1 pathologies.

Due to the excessive disease burden resulting from the two conditions, early HTLV-1 clinical trials with ARVs predominantly examined whether the drugs could improve the prognosis of patients with ATLL or alleviate debilitation imposed by HAM/TSP. However, since HTLV-1 persists via mitotic spreading and not de novo replicative infection (where ARVs antagonize replicating virus) following the acute stage of infection, the drugs were invariably found to be ineffective at treating either condition or reducing the HTLV-1 proviral load in carriers [43,44,45]. In a series of in vitro studies, including the current article, we set out to evaluate whether a panel of approved HIV drugs, INSTIs and NRTIs in particular, could be used for HTLV-1 prevention rather than therapeutics. The ultimate goal was to use any positive results to persuade pharmaceutical companies or governments in endemic areas to conduct clinical trials to assess the efficacy of these drugs as HTLV-1 pre-exposure prophylaxis (PrEP).

In this work, we demonstrate the anti-HTLV-1 activities of TAF and DTG in cell culture, confirming that all approved INSTIs and tenofovir prodrugs can potently inhibit HTLV-1 transmission [9,11]. The EMA and FDA already approve tenofovir prodrugs and the second-generation INSTI CAB as HIV PrEP. TDF-based PrEP entered the clinic a decade ago, leading to a dramatic fall in HIV incidence rates among vulnerable groups because strict adherence to the recommended daily dose is 99% effective at preventing infection. The most transformative advance in PrEP is probably the advent of long-acting CAB, which reduces the need for daily pill dosing to as few as six injections a year. There is no biological indication that current PrEP regimens would fail to protect at-risk persons from contracting HTLV-1, especially since HIV and HTLV-1 share both a primary cell target and transmission routes [46]. More crucially, the protein-adjusted ARV concentrations observed in the blood of healthy individuals taking oral tenofovir-based or injectable CAB-based HIV PrEP are several times the maximum doses deployed in our studies. Adopting ARVs for HTLV-1 prevention is especially advantageous because we know these drugs are safe in humans. Indeed, repurposing current ARVs is more ethical than developing novel HTLV-1-specific prophylactics, as the latter requires extensive preclinical evaluation. Moreover, the genomic stability of HTLV-1 relative to HIV-1 means there is a reduced risk of drug-resistant HTLV-1 strains emerging following the widespread adoption of ARVs as HTLV-1 PrEP [47]. 

Despite DTG being the backbone of most modern cART, no pill containing this INSTI is currently slated for use as HIV PrEP, since the current options are already highly effective, and pharmaceutical efforts are shifting to developing more long-acting agents. To this end, a recently developed intramuscularly administered long-acting form of DTG showed early promise in animal experiments, with pharmacokinetics suggesting that three-month dosing intervals with the prodrug could be possible [48]. 

Breastfeeding is the primary route for vertical (mother-to-child) transmission of HTLV-1 and is a leading indicator for ATLL development [49]. As such, interventions directed at the first year of life, including withholding breastmilk in favor of formula as implemented in Japan, may reduce the future incidence rates for the most mortal of HTLV-1-associated diseases. Screening mothers for HTLV-1 and feeding infants on formula are strategies that are currently exclusive to wealthy countries and would not be economically viable, culturally acceptable, or safe (when no access to clean water can be guaranteed) in other regions where HTLV-1 is endemic. Therefore, in the absence of a vaccine, PrEP may provide the only cost-effective option for stemming childhood infections and future caseloads of ATLL in lower–middle-income countries. If rolled out alongside campaigns to increase HTLV-1 awareness, clinically successful PrEP would drastically reduce sexual transmission rates, offering the first effective transmission barrier to condomless sex, through which 70% of new HTLV-1 cases currently occur [46,50]. Moreover, since sexual transmission is associated with myeloneuropathy prognosis, PrEP could prove an efficient measure in quelling HAM/TSP cases, too.

Since HTLV-1 persists mostly through mitotic spread following acute infection, it is unlikely administering ARVs to HTLV-1^+^ mothers can prevent vertical transmission to breastfeeding children, as seen for HIV, where an undetectable proviral load is the norm in mothers taking cART [46,51]. Therefore, although unfavorable, directly administering PrEP to neonates and infants born to HTLV-1^+^ mothers will be necessary. Regardless of the administered route of first-in-class HTLV-1 PrEP, daily oral medication or long-lasting injections, clinical trials to determine their safety and tolerable doses are required before their potential rollout in breastfeeding neonates and infants. 

It is crucial to be ready for unforeseen circumstances. Therefore, efforts to develop HTLV-1-specific compounds must continue in the unlikely scenario where INSTIs and tenofovir prodrugs fail as HTLV-1 prophylactics in vivo. Recent cutting-edge HIV-1 drugs to reach clinical trials do not target enzymatic active sites. Instead, allosteric inhibitors of integrase (ALLINIs) target an allosteric surface on the HIV-1 intasome, inducing IN multimerization and aberrant virion formation, whereas LEN tightly binds adjacent p24 monomers to prevent capsid disassembly [34,52,53]. Structures of deltaretroviral intasomes are now available, and an allosteric surface corresponding to ALLINIs interface may exist but is yet to be determined. The crystal structure of HTLV-1 p24, let alone the capsid lattice, is unresolved, so developing a capsid inhibitor via structure-guided methods is currently impossible. All in all, HTLV-1-specific structural antivirals are a long way from reaching clinical trials. 

## 4. Materials and Methods

### 4.1. Cell Lines

MT-2 and Jurkat E6.1 cells (obtained from ATCC, Manassas, VA, USA) were grown and maintained in a humidified atmosphere at 37 °C in RPMI medium (Gibco, Thermo Fisher Scientific, Waltham, MA, USA) supplemented with 10% fetal bovine serum (FBS, Sigma, Burlington, MA, USA), 100 U/mL penicillin, 100 μg/mL streptomycin, and 0.25 μg/mL Amphotericin B (Gibco). Human embryonic kidney (HEK) 293T cells were maintained in a humidified atmosphere at 37 °C in DMEM (Gibco) supplemented with 10% fetal calf serum (FBS), 1% non-essential amino acids (Gibco) and 1% penicillin/streptomycin (P/S) (Gibco). 

### 4.2. Structure Visualisation and Homology Modelling

All structural visualizations were performed in UCSF ChimeraX software v1.7.1 [54]. Predicted models of HTLV-1 RT and p24 monomers were generated using the AlphaFold 2 command tool on ChimeraX [55]. Amino acid alignment and homology modeling of the best model HTLV-1 RT to HIV-1 p66 were previously executed by Tardiota and colleagues [56]. Residue alignment of the best model of HTLV-1 p24 with HIV-1 p24 was determined after superimposition using rigid body alignment on ChimeraX. All chemical structures were downloaded from the public domain PubChem. The PubChem Compound Identification (CID) numbers of the drugs used in the study are TAF—9574768, RAL—54671008, BIC—90311989, DTG—54726191 and LEN—133082658.

### 4.3. Cell Treatment and Cell-to-Cell Infection of HTLV-1

A detailed protocol outlining in vitro cell culture infection with authentic HTLV-1 can be found in our earlier works [9,10,11]. Briefly, a day before infection, Jurkat cells were seeded in complete RPMI dosed with serial dilutions concentrations of TAF (8 µM, 4 µM, 2 µM, 200 nM, 20 nM, 2nM, 200 pM, 20 pM, 2 pM, 200 fM), RAL (20 µM, 2 µM, 200 nM, 20 nM, 2nM, 200 pM, 20 pM, 2 pM, 200 fM, 20 fM), DTG (800 nM, 80 nM, 8 nM, 800 pM, 80 pM, 8 pM, 800 fM, 80 fM, 8 fM, 0.8 fM) or LEN (100 nM, 33.33 nM, 11.11 nM, 3.7 nM, 1.23 nM, 0.41 nM, 0.14 nM), and the drug vehicle (DMSO). On the day of infection, MT-2 cells, which are persistently infected with and transmit HTLV-1, were exposed to a sub-lethal dose of gamma irradiation (400 Gray) and cocultured with Jurkat cells at a 1:1 ratio in serum-free medium supplemented with the drugs mentioned above. After 18 h, the coculture was washed with PBS, resuspended in depletion buffer (0.1% FBS, 2 mM EDTA in PBS), and gently tumbled (4 °C, 1 h) with anti-CD25^+^ magnetic beads (DynaBeads, Thermo Fisher Scientific) to remove MT-2 cells. Unbound Jurkat cells were maintained and then expanded in drug-supplemented complete RPMI for 12–14 days, after which genomic DNA was harvested for downstream analysis. Cell viability assays were carried out alongside infection assays to determine drug cytotoxicity. Jurkat cells were treated with matched concentrations of drugs as infected samples and maintained for 12–14 days before cell viability was determined by alamarBlue^TM^ (Thermo Fisher Scientific) following the manufacturer’s instructions.

### 4.4. Quantifying the HTLV-1 Proviral Load and Integration

The proviral load (PVL) was measured following a protocol outlined elsewhere [11,57]. In brief, the concentration of genomic DNA from infected Jurkat cells was determined by nanospectroscopy (DeNovix, Wilmington, DE, USA), and samples were diluted to 5 ng/μL for standardization. qPCR reactions to amplify HTLV-1 *tax* and human *ALBUMIN* gene products were performed using TaqMan^TM^ reagents (Thermo Fisher Scientific). The copy numbers of both genes were enumerated by comparison to standard curves generated from the patient-derived 11.50 T cell clone, which has a single known integration site. PVL was calculated by comparison of *tax* and *ALBUMIN* copy numbers as a fraction, multiplied by 100 to generate a percentile value assuming a single copy of tax and two copies of albumin per infected cell [58]. Data were normalized relative to DMSO-treated samples and fitted with dose–response curves in GraphPad Prism v 10.1.0. For samples treated with TAF, RAL and DTG, cells receiving the highest drug concentration, those near the inflexion point of PVL dose–response curves and DMSO-treated cells were subjected to Alu-PCR to amplify integrated provirus [59]. Provirus copy numbers were normalized to albumin, and DMSO-treated samples were arbitrarily set to 100%. A summary of primer sequences used in this study can be found in the supplementary table of Schneiderman et al., 2022 [11].

### 4.5. Production and Infection with HIV GFP Reporter Virus

To generate pseudoparticles, HEK 293T cells were co-transfected with three plasmids: an HIV (pCMV-∆8.91) packaging construct, a GFP reporter plasmid (pCH-GFPW) and an expression vector encoding vesicular stomatitis virus G protein (pCG-VSV-G) [60,61]. Supernatants containing HIV-1 pseudoparticles were collected at 48 and 72 h post-transfection and filtered. To measure the early-stage anti-HIV-1 activity of LEN, Jurkat cells were seeded in complete RPMI dosed with serial dilutions concentrations of LEN a day before infection. The following day, Jurkat was transduced with HIV-1 pseudoparticles for 4 h in serum-free RPMI in the presence of LEN. After this, the cells were washed in PBS, reseeded in complete RPMI containing LEN and expanded for three days. Infection was determined by flow cytometry on either the LSR II or LSR Fortessa machines (Becton Dickinson (BD) Biosciences, Franklin Lakes, NJ, USA), and data were analyzed using FlowJo 10.9 software (BD Biosciences). Percentage infectivity was normalized to DMSO-treated samples. To measure the effect on the drug at the late/egress stage of the viral life cycle, producer HEK 293T cells were maintained in DMEM containing LEN 24 h post-transfection onward. This pseudoparticle preparation was then used to transduce Jurkat cells for 4 h, and the rest of the experiment proceeded as outlined above. 

### 4.6. HTLV-1 Infection of Primary CD4^+^ T Cells by Co-Culture with MT2 Cells

The MT-2 cells were cultured for a minimum of 2 weeks in complete T cell media (RPMI medium supplemented with 5% human serum, 100 U/mL penicillin, 100 μg/mL streptomycin, 10 mM HEPES buffer solution and 1× nonessential amino acids) before experiments. On the day of the experiment, the MT-2 cells were irradiated with 400 Gray and then labeled with CellTrace CFSE (Thermo Fisher Scientific), as per the manufacturer’s instructions, to help distinguish MT-2 cells from primary CD4^+^ T cell populations by flow cytometry. 

On the day of infection, peripheral blood from uninfected volunteers was collected in sodium heparin tubes, and PBMCs were isolated immediately after blood draw by Ficoll Hypaque gradient centrifugation. CD4^+^ T cells were magnetically enriched by negative selection according to the manufacturer’s specifications (StemCell Technologies, Vancouver, BC, USA). The primary CD4^+^ T cells were treated with the desired dose of ARV for 3 h at a concentration of 1 million cells/mL in complete T cell media supplemented with 20 U IL-2 before MT-2 cells were added at a 1:4 ratio to the primary CD4^+^ T cells. The media were changed every 2–3 days with 20 U/mL IL-2 and the desired concentrations of the antiretroviral drugs until the cells were stained on day 12 of the experiment. 

Cells were stained with LIVE/DEAD Fixable Dead Cell Dyes (Thermo Fisher Scientific) according to the manufacturer’s specifications. An Fc receptor (FcR) blocking step was performed using FcR Blocking Reagent Human (Miltenyi Biotec, Cologne, Germany) before cell surface antibody staining. The antibodies used in the cell surface staining were the following: CD3 (UCHT1), CD4 (RPA-4), CD25 (M-A251) and CD8 (SK1). Cells were subsequently fixed using the Foxp3 staining buffer kit (Invitrogen, Waltham, MA, USA) following the manufacturer’s specifications. HTLV-1-infected cells were identified by staining for Tax (LT-4) [62]. Intracellular staining was performed using the Foxp3 staining buffer kit (Invitrogen). Following the staining, cells were resuspended in PBS for flow cytometry analysis. Samples were run on a Fortessa instrument (BD Biosciences) and analyzed using FlowJo v.10. Gating was performed to isolate single-cell populations, and then the LIVE/DEAD dye was used to select live CD3^+^ cells. The live cells were then analyzed for CFSE and CD4^+^ expression, with the CFSE-negative CD4^+^ cells being taken forward. The CD4^+^ population was then measured for CD25 and Tax expression and used for infection quantification.

Informed consent was provided by all participants before the blood draw. Blood samples were obtained through the Communicable Diseases Research Tissue Bank (NRES ID: 20/SC/0226). 

## 5. Conclusions

While the field awaits the discovery of HTLV-1 antivirals, a feat entirely dependent on sufficient funding, we may not need to reinvent the wheel, as the saying goes. This study is the latest in a series demonstrating that INSTIs and tenofovir prodrugs are similarly effective at preventing HTLV-1 and HIV transmission in cell culture (Table 1). We have provided the structural and mechanistic specifics underpinning conserved functionality for these drugs toward the distant orthoretroviruses, which should give confidence as to their potential use in preventing new cases of HTLV-1. Poor HTLV-1 awareness and a dearth of treatment options for the two major HTLV-1-associated pathologies necessitate a prophylactic measure. Our collective findings indicate safe, well-researched HTLV-1 prophylactics likely already exist in the form of ARVs. Indeed, adopting ARVs in endemic settings may be the most facile route to eliminating HTLV-1 transmission, a target recently set out by the WHO [63]. Therefore, we recommend that INSTIs and tenofovir prodrugs are urgently clinically evaluated as potential HTLV-1 PrEP. Failing this, the very least pharmaceutical companies can do is incorporate HTLV-1 incidence surveillance into ongoing HIV PrEP clinical trials in regions where HTLV-1 is endemic.

## Figures and Tables

**Table 1:** Overview of antiretrovirals’ efficacy in blocking HTLV-1 transmission in vitro.

ANTIRETROVIRAL	HTLV-1 EC_50_ (nM)	HIV-1 EC_50_ (nM)
**INSTI:**		
Raltegravir	6.42 ± 4.24 [9]	9.4 ± 1.4 [64]
Elvitegravir	9.57 ± 5.54 [9]	2.4 ± 0.9 [64]
Dolutegravir	0.25 ± 0.42	1.4 ± 0.3 [64]
Bictegravir	0.30 ± 0.17 [9]	1.6 ± 0.4 [64]
Cabotegravir	0.56 ± 0.26 [11]	1.5 ± 0.3 [65]
**NRTI:**		
Tenofovir disproxil fumarate	17.78 ± 7.16 [9]	50 ± 30 [66]
Tenofovir alafenamide	15.10 ± 9.06	5.1 ± 2 [67]

## Data Availability

Data are contained within the article.
